# The Structure of the Electric Double Layer of the Protic Ionic Liquid [Dema][TfO] Analyzed by Atomic Force Spectroscopy

**DOI:** 10.3390/ijms222312653

**Published:** 2021-11-23

**Authors:** Christian Rodenbücher, Yingzhen Chen, Klaus Wippermann, Piotr M. Kowalski, Margret Giesen, Dirk Mayer, Florian Hausen, Carsten Korte

**Affiliations:** 1 Institute of Energy and Climate Research (IEK-14), Forschungszentrum Jülich GmbH, 52425 Jülich, Germany; y.chen@fz-juelich.de (Y.C.); k.wippermann@fz-juelich.de (K.W.); c.korte@fz-juelich.de (C.K.); 2 Institute of Energy and Climate Research (IEK-13), Forschungszentrum Jülich GmbH, 52425 Jülich, Germany; p.kowalski@fz-juelich.de; 3 Jülich Aachen Research Alliance, JARA Energy & Center for Simulation and Data Science (CSD), 52425 Jülich, Germany; 4 Peter Grünberg Institute (PGI-6), Forschungszentrum Jülich GmbH, 52425 Jülich, Germany; m.giesen@fz-juelich.de; 5 Institute of Biological Information Processing (IBI-3), Forschungszentrum Jülich GmbH, 52425 Jülich, Germany; dirk.mayer@fz-juelich.de; 6 Institute of Energy and Climate Research (IEK-9), Forschungszentrum Jülich GmbH, 52425 Jülich, Germany; f.hausen@fz-juelich.de

**Keywords:** fuel cells, electrolytes, ionic liquids, electric double layer

## Abstract

Protic ionic liquids are promising electrolytes for fuel cell applications. They would allow for an increase in operation temperatures to more than 100 °C, facilitating water and heat management and, thus, increasing overall efficiency. As ionic liquids consist of bulky charged molecules, the structure of the electric double layer significantly differs from that of aqueous electrolytes. In order to elucidate the nanoscale structure of the electrolyte–electrode interface, we employ atomic force spectroscopy, in conjunction with theoretical modeling using molecular dynamics. Investigations of the low-acidic protic ionic liquid diethylmethylammonium triflate, in contact with a platinum (100) single crystal, reveal a layered structure consisting of alternating anion and cation layers at the interface, as already described for aprotic ionic liquids. The structured double layer depends on the applied electrode potential and extends several nanometers into the liquid, whereby the stiffness decreases with increasing distance from the interface. The presence of water distorts the layering, which, in turn, significantly changes the system’s electrochemical performance. Our results indicate that for low-acidic ionic liquids, a careful adjustment of the water content is needed in order to enhance the proton transport to and from the catalytic electrode.

## 1. Introduction

Polymer membranes doped with Brønsted acidic protic ionic liquids (PILs) are considered promising electrolytes for future energy-efficient intermediate-temperature polymer electrolyte fuel cells (IT-PEFCs) [1,2,3,4]. The high stability and high proton conductivity of PILs, even at elevated temperatures, would enable a fuel cell to be operated above 100 °C [5,6,7]. This would facilitate heat and water management and could therefore be beneficial, in particular, for emission-free automotive applications. In order to select suitable ionic liquid electrolytes, their electrochemical properties must be understood in greater detail [8]. Ionic liquids are essentially salt melts consisting of bulky ions; therefore, electrostatic interactions as well as steric effects determine the arrangement of the ions in the bulk phase and at the interface to a catalytic electrode [9,10,11,12]. Hence, the structure of the electrolyte–electrode interface differs from that of conventional (diluted) aqueous solutions [13,14,15,16,17,18,19]. In consequence, the electrochemistry determining the kinetics of the interface processes also differs [20]. Instead of a classical aqueous double-layer structure as described by Helmholtz, Gouy-Chapman, and Stern models, respectively, the interface layer of ionic liquids consists of a dense layering of alternating anion and cation layers [21]. Many experimental studies on the interface structure have been conducted using various methods, with atomic force spectroscopy (AFS) being one of the most direct of these [15]. By approaching and retracting a tip immersed in the ionic liquid to and from an electrode, a characteristic dense layering has been found with an extension of several nanometers into the bulk [22]. The structure of the interface layer is also influenced by the presence of water [23,24]. Depending on the hydrophilicity of the ionic liquids, water can be incorporated from the surrounding atmosphere [25]. During fuel cell operation, the ionic liquids reach a certain steady-state water concentration, as H_2_O is produced by the reaction between hydrogen and oxygen [26]. In many ionic liquids, it has been found that water leads to distortion and weakening of the interface structure [27,28]. This has been attributed to the preferential adsorption of water on the electrified electrode, as well as to the interaction between the H_2_O molecules and ions of the PIL via hydrogen bonds [29]. However, in some ionic liquids, the presence of water was found to be a prerequisite for the formation of a detectable structured interface layer [30]. This indicates that the nanoscale-structure of ionic liquids is highly complex and must be analyzed in detail for each class of ionic liquid.

**Figure 1:**
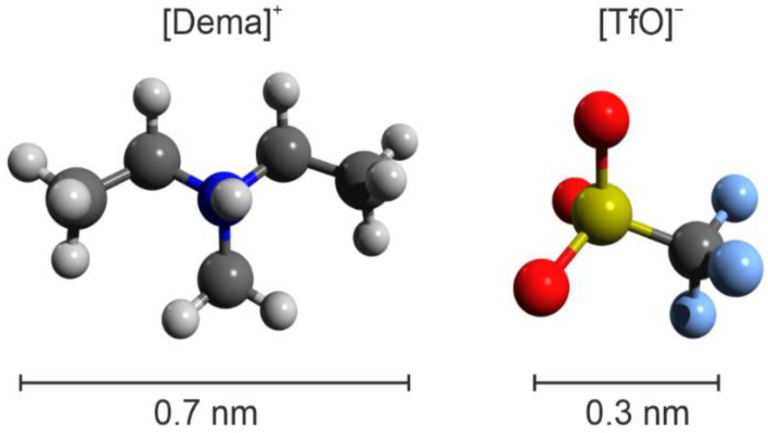
Molecular structure of the investigated protic ionic liquid [Dema][TfO].

In this study, we focus on the investigation of the double-layer structure of protic ionic liquids that have potential for fuel cell applications. As a model material, we selected diethylmethylammonium triflate ([Dema][TfO]), which is a non-corrosive room-temperature ionic liquid that has already proven its applicability as an electrolyte for fuel cells and electrolysis [6,31,32]. The molecular structures of the [Dema]^+^ cation and the [TfO]^−^ anion are shown in Figure 1. In contrast to highly acidic PILs, the exchange rate of protons between the cations and water in [Dema][TfO] is very slow. There exists a vehicular bulk transport mechanism for protonic charge carriers, i.e., the [Dema]^+^ cations, and no transition to cooperative transport with increasing water content has been observed [33,34]. Nevertheless, a significant correlation between the water content of [Dema][TfO] and the oxidation of the platinum (Pt) surface has been found, which has been attributed to the structure of the electric double layer [35]. Herein, we combine atomic force spectroscopy and atomistic simulations in order to investigate the structure of the double layer at different potentials and water contents.

## 2. Results

**Figure 2:**
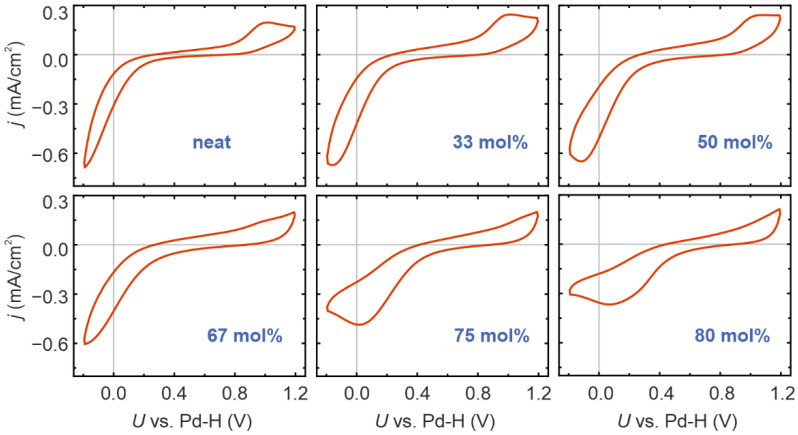
Cyclic voltammetry of [Dema][TfO] with different water contents in contact with Pt electrodes under an oxygen atmosphere. The measurements were performed at room temperature.

In order to obtain an overview of the influence of water on the electrochemical performance of the PIL [Dema][TfO], we performed a cyclic voltammetry (CV) on six mixtures with different water contents ranging from “neat” to 80 mol% (Figure 2). In agreement with previous CV investigations on protic ionic liquids [31,36,37], three characteristic potential regions can be identified. The measurements were conducted under an oxygen atmosphere; therefore, the high negative current density at potentials below 0.4 V, measured vs. a Pd–H quasi reference electrode, relates to the oxygen reduction reaction (ORR). The intermediate potential range between 0.4 V and 0.8 V can be identified as the double layer region in which the ordering and charging of the PIL/Pt interface occurs. In this potential range, a mainly capacitive current flows, with almost no faradaic current. At higher potentials, a peak at approximately 1 V can be observed; this is associated with the discharge of H_2_O molecules and the electrosorption of oxygen on the electrode, which leads to the formation of PtO_x_ [36]. The addition of water significantly changes the shape of the CV curves. The anodic peak around 1 V initially increased until 33 mol%, then remained almost constant up to 50 mol% before decreasing for higher water contents. 

It can also be observed that, with increasing water content, the onset potential of the cathodic ORR processes shifted to higher values, indicating that the activation overpotential decreased and the proton transport to the interface became more efficient. As [Dema][TfO] has a very low acidity, the amount of H_3_O^+^, which acts as an additional proton donor, is relatively low in the PIL/water mixture (10^−5^ to 10^−6^ mol/L) [34]. Hence, it can be assumed that this effect is not the main cause of the shift in the onset potential, but that the addition of water changes the structure of the PIL, particularly in the region of the double layer. Here, the hydration of ions and the build-up of networks of hydrogen bridges has a significant influence on the activated complex, as well as the charge and proton transfer at the interface. Furthermore, the cathodic peak is also partially determined by the reduction of Pt oxide and the adsorbation of hydroxy groups (OH) on the Pt surface. This becomes particularly obvious in the CV curves at a water content above 75 mol%, whereby the reduction peak shifts to positive potential and clearly superposes the ORR peak.

**Figure 3:**
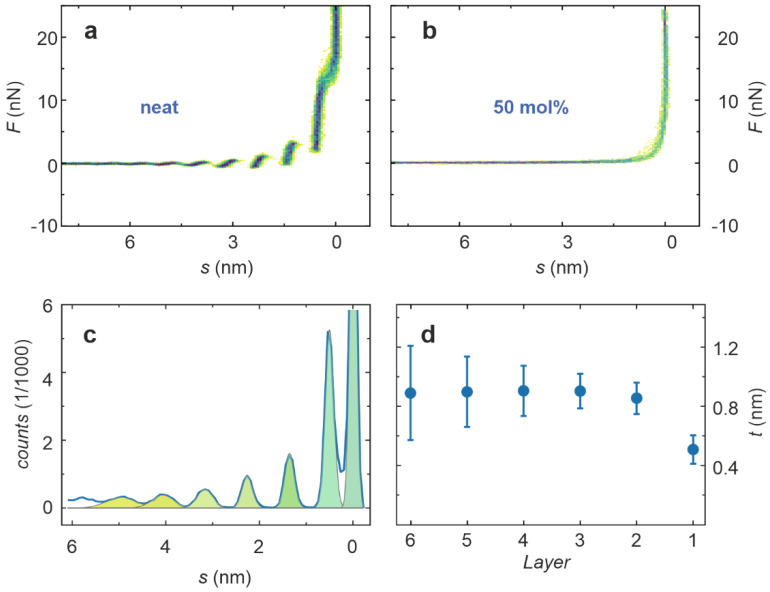
Force–separation curves obtained on the Pt–[Dema][TfO] interface during approaching. (**a**) 2D histograms of 50 curves measured on neat [Dema][TfO] and (**b**) a 1:1 mixture of [Dema][TfO] and water; (**c**) separation histogram calculated from the approach curves of neat [Dema][TfO]; and (**d**) thickness of the layers calculated from the peak fitting of the separation histogram.

In order to gain insight into the interface formed between [Dema][TfO] and Pt by a direct method, atomic force spectroscopy was employed. Figure 3a shows 2D histograms of 50 subsequently recorded force–separation curves from the approaching of the Pt surface in neat [Dema][TfO], without an applied voltage. The fluctuations between the curves were relatively low, and a high reproducibility in force curve measurements could be achieved. Characteristic steps can be identified, indicating that a dense layering in the PIL at the interface, ranging up to six nanometers into the liquid, was present. In contrast to this, the measurement in a mixture of [Dema][TfO] and 50 mol% water did not reveal any layers, verifying that the dense structure is dissolved by the presence of water. 

**Figure 4:**
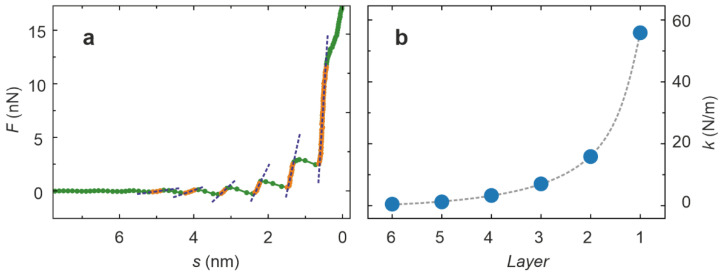
Mechanical analysis of the interface layers. (**a**) Linear regression of the regions of the average force–separation curve with a positive slope; (**b**) resulting stiffness of the layers (the error bars are smaller than the blue dots).

In order to analyze the thickness of the layers of neat [Dema][TfO], a histogram of the separation values of the approach curves was generated (Figure 3c). Apart from the peak at zero separation, presumably marking the Pt surface, six peaks can be clearly identified. These were simulated by Gauss functions, whose positions were used to calculate the thickness of the layer. The result displayed in Figure 3d reveals a thickness *t* in the range of 0.9 nm for most of the layers, which approximately corresponds to the dimension of a [Dema][TfO] ion pair. The innermost layer only had a thickness in the range of 0.5 nm, which indicates a close interaction between the Pt surface and the adsorbed molecules, leading to a dense packaging. These measurements were performed without an applied external voltage, corresponding to a slightly positive electrode potential, as a positive open circuit voltage was present. Hence, it can be assumed that the TfO cations primarily form the first molecular layer, being preferentially attracted by the positively charged electrode. When approaching the electrode surface by the tip, the force necessary to penetrate each layer increases with decreasing separation, revealing that the packaging and charge ordering becomes more pronounced closer to the interface. In order to quantify this effect, the average of the approach curves in Figure 3a was calculated and the sections showing a positive slope were selected and fitted by linear regression as illustrated in Figure 4a. The slope of the regression curves corresponds to the stiffness of the layers displayed in Figure 4b. It can be seen that the stiffness increases from a few N/m for the outermost layers to a value above 50 N/m for the layer that was closest to the interface. This confirms the existence of a dense and ordered packaging in the PIL at the interface, which becomes weaker with increasing distance from the interface.

**Figure 5:**
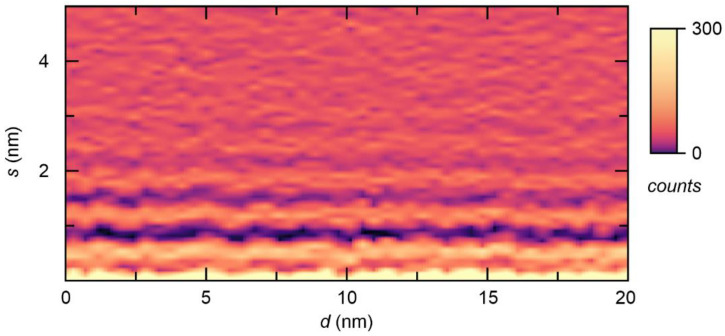
Separation histogram of a series of 50 force–separation curves recorded along a line of 20 nm revealing the dense layering across the entire surface.

In order to obtain a first overview of the spatial structure of the interface, a series of 50 force–separation curves was recorded along a line with a length of 20 nm. For each curve, a separation histogram was calculated, which is displayed as a 2D plot in Figure 5. The local maxima and minima of all separation histograms can be found at roughly the same position relative to the interface, which indicates that the dense layering was present at the entire interface. No significant distortions in the structure could be observed. As we used an epi-polished Pt crystal with an atomically flat surface, this behavior is unsurprising. When employing a rough surface, as would be the case for a real fuel cell electrode, local structural distortions might also become relevant. For example, it was observed for aprotic ionic liquids in contact with graphite that topological defects in the layered structure could occur above the step edges of the surface [38].

**Figure 6:**
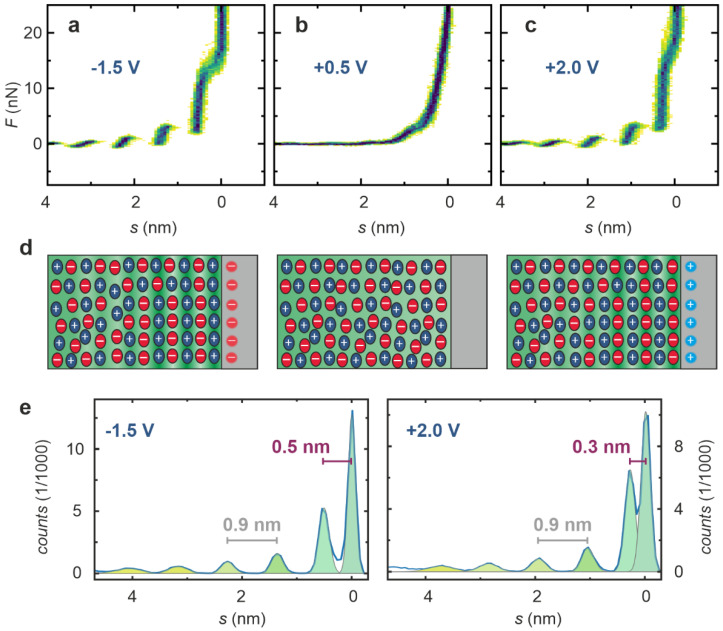
Influence of the electrode voltage on structure of the interface between Pt and neat [Dema][TfO]. 2D histograms of 50 force–separation curves measured during the tip approach at voltages of (**a**) −1.5 V, (**b**) +0.5 V, (**c**) +2.0 V (note that this voltage was not measured versus a reference electrode); (**d**) schematic illustration of the ordering in the ionic liquid at the interface; (**e**) separation histograms for −1.5 V and +2.0 V.

As for understanding the performance of a PIL in a fuel cell, for which interaction with a charged electrode is relevant, we measured force–separation curves after applying a voltage to the electrode. In Figure 6, three 2D histograms obtained with different voltages are compared. At a negative voltage of −1.5 V (Figure 6a), distinct steps can be observed that are slightly more pronounced than in the case without the presence of the applied voltage (cf. Figure 3). As is shown in the schematic illustration in Figure 6d, the first ion layer would consist of cations, as the surface is negatively charged. It must be noted that due to technical limitations, no reference electrode was employed and the voltage applied between the Pt electrode and the grounded stainless steel holder of the microscope was not equal to the potential applied during the CV measurements depicted in Figure 2. When the voltage was increased to +0.5 V, the force–separation curves changed significantly and almost no steps were present (Figure 6b). This indicates that this voltage corresponds to the potential of zero charge (PZC), at which the electrostatic interaction between the electrode and the ions of the liquid is the lowest, and the interface region is unstructured. Upon increasing the voltage to +2 V, the characteristic steps in the force–separation curves can again be seen (Figure 6c), revealing that the ordering in the interface region was reestablished. As the voltage was reversed compared to the situation in the beginning, it can be assumed that the anions now formed the first layer at the interface, as illustrated in Figure 6d. The force–separation curves measured at −1.5 V and +2.0 V were further analyzed by calculating separation histograms and performing peak fitting as shown in Figure 6e. At a negative potential, the distance between the surface and the first detected step in the curves was 0.5 nm, significantly larger than the corresponding distance of 0.3 nm at a positive potential. This gives further evidence that at a negative electrode potential, the first layer consists of the larger cations, while under positive potential, the first layer is an anion layer. The fact that these significant differences are measured also indicates that the force was sufficient to reach the Pt surface and that the steep increase in the force curves really marks the position of zero separation between tip and electrode. The distances between the subsequent steps further away from the interface were still in the range of 0.9 nm for both polarities, corresponding to the dimension of an ion pair.

**Figure 7:**
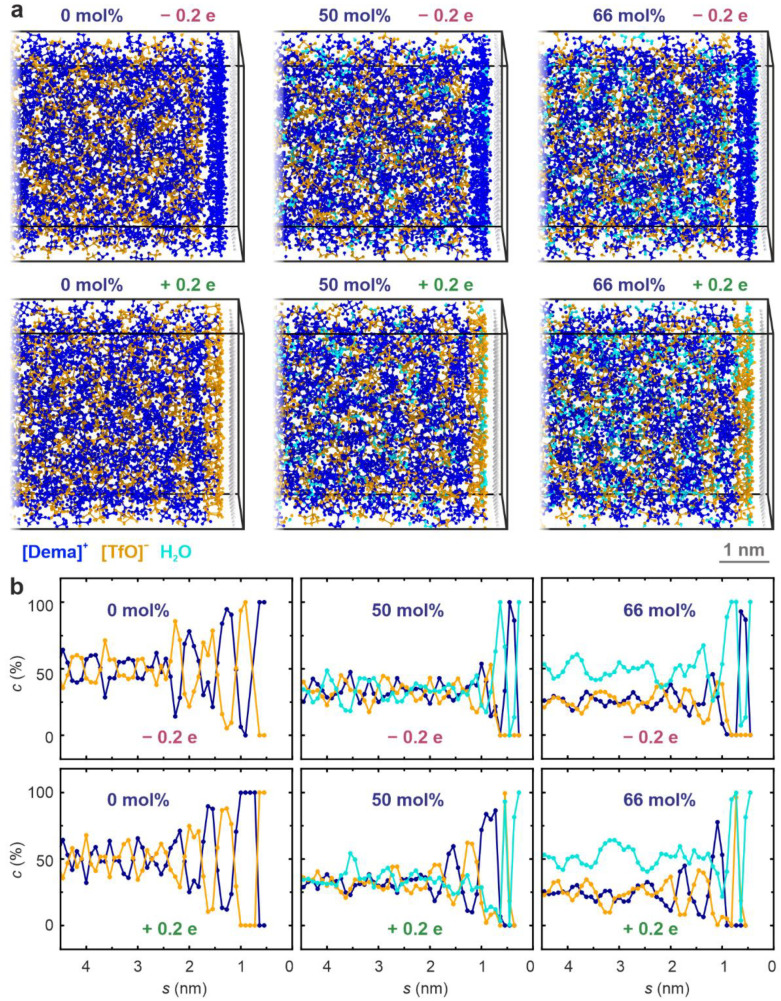
Molecular dynamics simulation of [Dema][TfO] with different water contents between two inversely charged Pt (100) electrodes: (**a**) snapshots of the molecular configurations in the interface regions after 5 ns of constant-volume simulation; (**b**) concentrations of anions, cations, and water as a function of the separation from the positrode and negatrode, respectively.

In order to illustrate the molecular movements that lead to the evolution of the double-layer structure, molecular-dynamics simulations were performed. Water-free [Dema][TfO] and PIL/water mixtures with water contents of 50 mol% and 66 mol% were simulated. A capacitor-like geometry with two oppositely charged electrodes was simulated. A charge of 0.2 e was assigned to the topmost atoms of the electrode. This value was estimated using the typical double layer capacitance of PILs and would correspond to a voltage of a few volts (for details, see Methods section). Figure 7a displays snapshots of the positrode and negatrode regions, respectively, after a 5 ns production run at room temperature. It can be clearly seen that the [Dema] cations (blue) form the first molecular layer at the negatrode, whereas the [TfO] anions (orange) accrue at the positrode. With increasing water content, this tendency persists, but a disordering of the structure with water molecules (light blue) being present in the double layer can already be identified in the snapshots. For a closer inspection of the simulated double-layer structure, the concentration of the molecules close to the electrodes was calculated by means of histograms of the center of mass position for each molecular species. The concentration–separation plots shown in Figure 7b indicate that the double-layer ordering with alternating anion and cation layers is most pronounced for the water-free PIL. The distance between subsequent layers of the same ion species is in the range of 0.7–0.8 nm, which is only slightly smaller than the experimental value measured by AFS, proving that the steps in the force–separation curves indeed correspond to layers of ion pairs. With increasing distance from the electrode, the excess of anions and cations in the layers decreases, approaching the stoichiometric concentration of 50% after several nanometers. This simulated transition from a dense layering to a diffuse double-layer structure could explain the results from atomic force spectroscopy revealing a decrease in stiffness with increasing separation from the interface (cf. Figure 4). The simulations of the [Dema][TfO]–water mixtures reveal that the presence of water significantly changes the structure of the double layer. It can be seen that the relative difference in anion and cation concentration in the layers is reduced by comparison to the water-free case. For the simulation of a water concentration of 66 mol%, the stoichiometric ratio (i.e., an equal anion and cation concentration of 33 mol%) is almost reached at a distance of 1–2 nm from the surface, resembling the classical double-layer behavior of aqueous electrolytes. Furthermore, it can be seen that water molecules are also present within the double layer and close to the electrode surface. Hence, it can be expected that the presence of water significantly influences the electrode reactions in a fuel cell, e.g., distorting the layered interface structure, acting as a proton donor and forming an oxide or hydroxide layer at the interface.

## 3. Discussion

Our experimental and theoretical results reveal that the protic ionic liquid [Dema][TfO] forms a dense layered interface in contact with a charged Pt electrode. The structure of the interface depends on the applied voltage and the water content in the liquid. With increasing water content, the double layer becomes distorted and a transition from the layered PIL behavior to that of a classical aqueous double layer occurs. Our findings illustrate that the structure of the double layer of ionic liquids is complex and differs significantly from aqueous solutions. Here, the layering effect, which has been observed for many different ionic liquids previously, is confirmed for the technologically relevant combination of the protic ionic liquid [Dema][TfO] and a catalytic Pt surface. Our nanoscale investigations provide valuable information about the double-layer structure, which is needed to improve the performance of future intermediate-temperature fuel cells, in particular with respect to the oxygen reduction reaction. We demonstrate that using a combination of different methods, an identification of the anion and cation layers in the interface region is possible. There is a strong dependence of the double-layer structure on the electrode potential, which can be monitored on an atomistic scale. Similarly, the water content has been identified as a key parameter for the morphology of the double-layer structure. Water contributes to the oxygen reduction reaction not only by acting as a proton donor but also by distorting the double-layer structure, e.g., due to the formation of hydrogen-bonded networks adsorbed on the Pt surface. With respect to applications of the electrolyte in potential fuel cells, our findings hint at the fact that a low-acidic PIL alone may not have promising potential, but that the mixture with water or with a second PIL as a proton donor could significantly enhance its performance [39]. Hence, it could be possible to tune the reactivity of PILs by means of controlled double-layer engineering.

## 4. Materials and Methods

Diethylmethylammonium triflate ([Dema][TfO], CAS No.: 945715-39-9) with a nominal purity of 98 wt% (IoLiTec, Heilbronn, Germany) was used. The initial water content of “neat” ionic liquid was in the range of 0.2 to 0.25 wt%, as determined by Karl Fischer titration. By mixing the ionic liquid with Milli-Q water, samples with different water content could be prepared.

Cyclic voltammetry (CV) was performed in a custom-made electrochemical cell as described by Wippermann et al. [2]. A Pt wire with a 1 mm diameter (99.95%, Goodfellow GmbH, Hamburg, Germany) was employed as the working electrode. A Pt crucible containing the electrolyte was used as the counter electrode and Pd–H served as a reference electrode. The measurements were conducted at room temperature under an oxygen atmosphere using a Zennium potentiostat (Zahner-elektrik, Kronach, Germany). For each water content, 10 cycles between −0.2 and 1.2 V were recorded with a rate of 0.1 V/s, and the tenth cycle was chosen for detailed analysis. 

Atomic force spectroscopy was performed using a Cypher atomic force microscope (Asylum, Santa Barbara, CA, USA) equipped with a droplet cell. An epi-polished Pt (100) single crystal with the dimensions of 10 × 10 × 1 mm^3^ (Mateck, Jülich, Germany) served as the working electrode and the stainless-steel tip holder as the counter electrode. In order to obtain a clean Pt surface, the crystal was annealed in a butane flame for one minute immediately before transfer to the measurement chamber. Au-coated Si tips (Tap150GD-G, Budget Sensors, Sofia, Bulgaria) were used to perform force spectroscopy while approaching the surface. Their resonance frequency, which was used to determine the force constant, was obtained by fitting the main peak in the thermal excitation spectrum. The typical force constant was in the range of 5 N/m. Force–distance curves were performed at an approach speed of 30 nm/s. To convert the measured force–distance curves into force–separation ones, the cantilever deflection, which was determined on the bare Pt surface in air, was subtracted [22]. For the sake of comparability, the force measured far away from the sample was set to zero, and the point of zero separation was set such that the curves overlapped in the region of the steep force increase at the surface. We assume that this region marks the Pt surface as no further steps were observed, even when increasing the force to 35 nN. Measurements were conducted without an applied voltage and for different voltages applied between the Pt crystal and the grounded cantilever holder. For each set of measurements, 50 force–separation curves were recorded and analyzed as 2D histograms.

Simulations by means of molecular dynamics were conducted using the LAMMPS code [40,41]. The [Dema][TfO] molecules were described by the OPLS all-atom force field [42]. The force field parameters were drawn from Nasrabi and Gelb [43]. Water was described by the SPC/Fw force field proposed by Wu et al. [44]. The interaction between the atoms in the liquid and the Pt surface was approximated by taking the Lennard–Jones potential of Pt [45] and applying the geometric mixing rule. A capacitor-like geometry was used with 450 ion pairs placed between two Pt (100) electrodes modeled by four atomic layers each. Mixtures of [Dema][TfO] and water with 50 mol% and 66 mol% were simulated by 407 ion pairs with 407 water molecules and 306 ion pairs with 720 water molecules, respectively. Periodic boundary conditions were set in the x and y directions, while in the z direction, a fixed boundary was simulated. The slab correction was applied, and the atoms of the electrodes were treated as immobile. The electrodes had a lateral dimension of 39 Å × 39 Å, and the distance between them was 83 Å. This value was chosen to allow for the formation of a bulk-like structure in the center between the electrodes, and is a good tradeoff between the computational costs and accuracy of results. Furthermore, the dimensions of the simulation cell were adjusted to reproduce the density of the bulk; this was determined by simulations of the pure ionic liquid in the NPT (constant particle number, pressure, and temperature) ensemble, yielding a density of 1347 kg/m^3^, which is close to the experimental value of 1294 kg/cm^3^ measured by Merkel et al. for the neat liquid [46]. With increasing water content, the distance of the electrodes was adjusted accordingly. The charging of the electrode was simulated by the constant charge method [47] assigning a charge of +0.2 e to all atoms in the terminal atomic layer of the bottom electrode, and a charge of −0.2 e to all atoms in the terminal atomic layer of the top electrode. As the electrical double layer capacity of protic ionic liquids has been found to be in the range of 50 µF/cm^2^ [2], the assumption of this charge is a reasonable approximation for a voltage of a few volts. In this way, the simulations can provide a first impression of the double-layer structure; however, the details of the electrolyte–electrode interaction, which involves local polarization, charge transfer mechanisms, proton-hopping, formation of oxide layers, and chemical reactions were neglected [48,49,50]. The initial structure of the ionic liquid between the molecules was generated by the Packmol program [51]. An equilibration step of 100 ps at 800 K and of 1 ns at 298 K was performed in the NVT (constant particle number, volume, and temperature) ensemble with a Nosé–Hoover thermostat before switching on the charging of the electrodes. In order to speed up the ordering of the molecules in the double layer, the equilibration step of 100 ps at 800 K was repeated before a 5 ns production run was started, with a timestep of 1 fs. The simulations were performed using the booster module of the Jureca supercomputer. 

## Data Availability

Data are contained within the article.
